# Venous Bubble Trap - Management Types During Minimal Invasive Extracorporeal Circuits

**DOI:** 10.21470/1678-9741-2020-0448

**Published:** 2023

**Authors:** Ignazio Condello

**Affiliations:** 1 Department of Cardiac Surgery, Anthea Hospital, GVM Care & Research, Bari, Italy. E-mail: ignicondello@hotmail.it

The use of minimally invasive extracorporeal circuits (MiECC) in cardiac surgery is expanding. In this letter to the Editor, I emphasize the air evacuation management from bubble trap through various strategies. The prevention and management of macro and micro gas embolism during minimally invasive extracorporeal circulation (ECC) is crucial. In particular, according to the classification of the Minimal Invasive Extracorporeal Technologies International Society position paper, the types of MiECC II, III, and IV are equipped with bubble trap in the venous line. The improved biocompatibility ameliorates organ preservation and reduces transfusion of homologous blood products^[1]^. However, the MiECC system is a closed-loop system, employing kinetically assisted venous drainage, which may increase the risk of introducing venous air. Venous air travels easily through a bypass system resulting in gaseous microemboli in the arterial line prior to entering the patient’s arterial circulation. The number of cerebral microemboli increases in conventional ECC systems during drug bolus injections, blood sampling, low blood volume levels in the venous reservoir, and infusions^[2]^. Microemboli activate the inflammatory response and may even obstruct the blood flow in the capillary vessels, causing ischemia of the tissues^[3]^. Consequently, these pathophysiological processes may lead to a decline in the cognitive function of the patient. Less microemboli were detected in the arterial line and in the cerebral vessels during the use of MiECC systems as compared to conventional systems. However, more recently, a study on the use of a customised MiECC system was terminated prior to study completion due to venous air leakage^[1]^. Others have also raised their concerns over patient safety by using a miniaturized ECC system, without a safety feature to remove venous air^[4,5]^. The use of an air removal device at the venous side of the MiECC system could avoid air entering this system and may increase patient safety. A venous bubble trap (VBT) was designed as air removal device for air separation in the venous line of MiECC systems and it was also designed for air separation in the venous line of minimized ECC circuits. The VBT was interconnected in the venous line of the MiECC system, and the air-removal was accomplished by a combination of two principles, centrifugal flow and bubble buoyancy. The 175-mm mesh screen separates air from blood and enables removal of trapped air from both sides of the screen through the evacuation port. Many perfusionists add an accessory bubble trap in the intracavitary aspirator (vent) line before the main bubble trap and air evacuation port is connected to the autotransfusion system, allowing to block the air from the vent before the VBT. Other circuit designs for MiECC have the vent line directly on the main VBT, the air evacuation port is connected to the autotransfusion system, or dynamic port connected to the roller pump head. In the cardiopulmonary bypass consoles, there are automated systems for air purge control, detected through ultrasound sensors from the venous line and bubble trap that activate the autoclamp and purge line.
